# Provision of and trust in COVID‐19 vaccines information: Perspectives of people who have had COVID‐19

**DOI:** 10.1111/hex.13706

**Published:** 2023-02-03

**Authors:** Laura Schackmann, Karin Hek, Marcia Vervloet, Ellen S. Koster, Liset van Dijk

**Affiliations:** ^1^ Nivel Netherlands Institute for Health Services Research Utrecht The Netherlands; ^2^ Unit of PharmacoTherapy, ‐Epidemiology & ‐Economics, Groningen Research Institute of Pharmacy University of Groningen Groningen The Netherlands; ^3^ Department of Pharmacoepidemiology & Clinical Pharmacology (UPPER) Utrecht University Utrecht The Netherlands

**Keywords:** choice to vaccinate, COVID‐19 people, COVID‐19 vaccines, information provision, trust

## Abstract

**Aim:**

The aim of this study was to understand the provision and need, quality of and trust in COVID‐19 vaccines information from the perspectives of people who have had COVID‐19 infection.

**Method:**

People who have had a COVID‐19 infection were approached via their general practice and invited to participate in the Nivel Corona Cohort. They completed questionnaires at baseline (Q1), and at three months (Q2). Outcome measures were based on health information‐seeking behaviour, as used in the Comprehensive Model of Information Seeking. Antecedents (i.e., gender, age, education level, health literacy) were used from Q1, and one's beliefs and experiences (i.e., trust in the information and healthcare system, how applicable the information is), information carrier factors (i.e., information quality perceptions and via which sources), health‐information seeking actions (i.e., decision to vaccinate and information sufficiency) and vaccination status from Q2. Data were analysed using descriptive analyses, analysis of variance tests (F‐tests) and *χ* ^2^ tests with the statistical software STATA.

**Results:**

Of the respondents (*N* = 314), 96% were vaccinated at least once, mostly after having had the virus. Most retrieved information about COVID‐19 vaccines on the website of the National Institute for Public Health and the Environment (79%), broader via the internet (56%), or from family and friends (35%). Almost all had trust in the information (89%) and healthcare system (94%). Most found the information applicable to their situation (67%). Moreover, most perceived the information as correct (71%) and did not perceive the information to be misleading (85%), while fewer people found the information reliable (59%) and clear (58%). Overall, the majority indicated that the information met their expectations to make a well‐informed decision to vaccinate (89%).

**Conclusion:**

Different characteristics of people who had COVID‐19 and sought information were identified, which is important to offer tailored information. People who had COVID‐19 in this study, mainly middle‐aged, vaccinated and highly educated, were generally positive about the vaccines information, but overall the reliability and clarity could be improved. This is important for a high vaccination uptake, booster programs and coming pandemics.

**Patient or Public Contribution:**

The questionnaire was reviewed by patients who had COVID‐19, one of whom is a health services researcher.

## 1 INTRODUCTION

Reliable and clear information is important for a high vaccination uptake,^1^ including booster programs and pandemics. To understand the provision of vaccines information and trust therein, the coronavirus disease 2019 (COVID‐19) is used as a case study. An effective way to tackle the COVID‐19 pandemic is through mass vaccination globally, though acceptance of the vaccines is often a major challenge.^2^ The COVID‐19 pandemic is a unique situation, where vaccines were developed more rapidly than usual. In the beginning, this may have had a negative impact on the trust that people had in the developed vaccines and could, therefore, negatively influence the choice to vaccinate.^3^, ^4^ Over the course of time, people have chosen to vaccinate, though, now with the emerging strains of COVID‐19 and the need for boosters,^5^ provision of information and trust therein are key aspects in being well‐informed to decide to vaccinate.

Specifically, the provision of COVID‐19 vaccines information and trust therein amongst people who have COVID‐19 is of interest. At the start of the COVID‐19 pandemic, it is likely that there is a high‐risk perception experienced by people as it is a new disease.^6^, ^7^ Preventative measures, such as vaccination programs, are steered by risk perception, as trends also show that uptake of the preventive measures was higher at the beginning of the pandemic than later on. As time passes, this risk perception may decrease as there is more information and experiences with the virus. People who have had COVID‐19 may have a lower risk perception as they have had the virus and may be less inclined to vaccinate, or instead, they may be more keen to vaccinate as they have experienced the virus severely and want to prevent being infected again. Hence, it is of interest to investigate this group's information‐seeking behaviour to tailor future vaccination campaigns, or the necessary COVID‐19 boosters due to the emergence of new strains.

**Figure 1:**
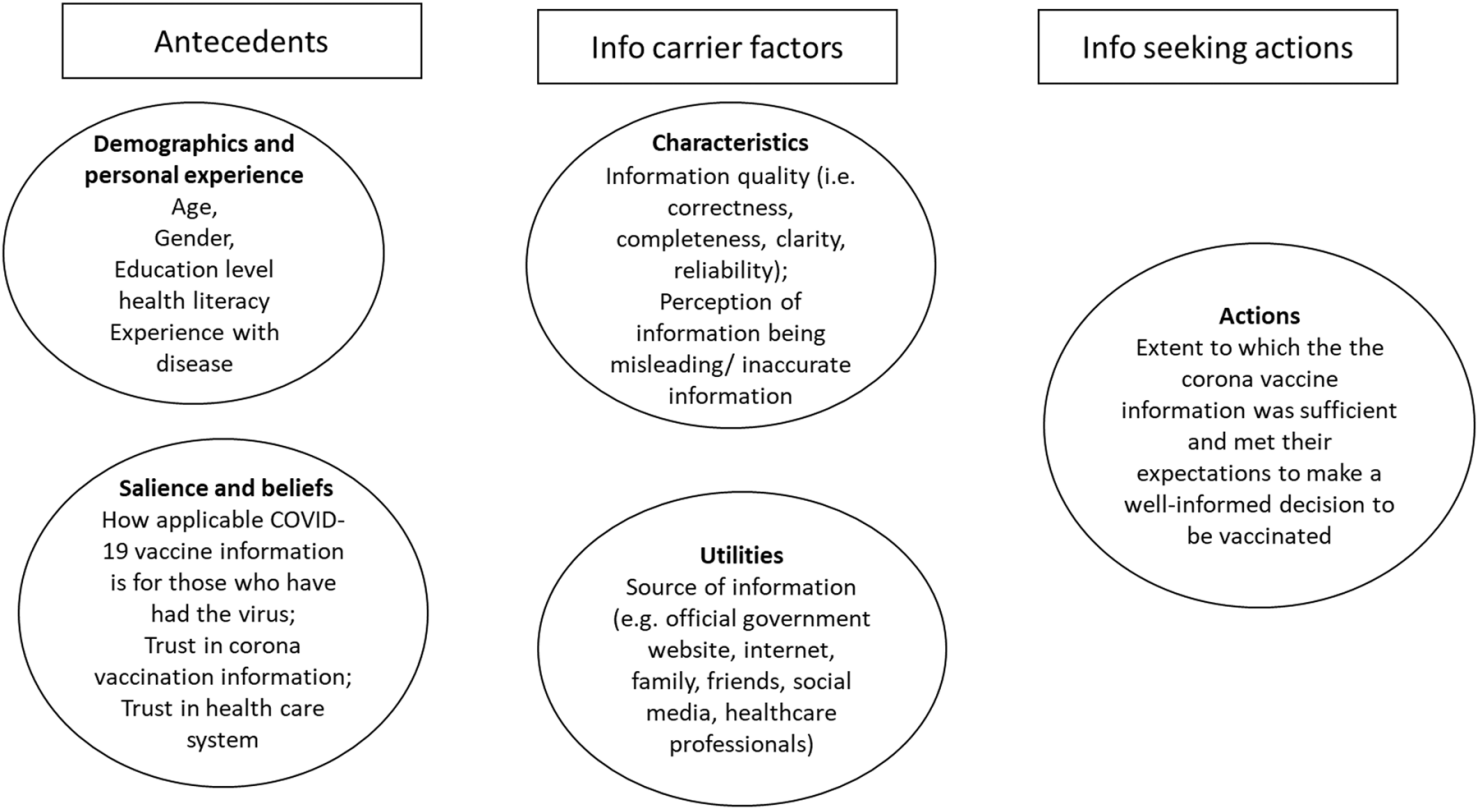
CMIS adapted to fit the context of COVID‐19 vaccines information‐seeking behaviour. CMIS, Comprehensive Model of Information Seeking; COVID‐19, coronavirus disease 2019.

There are various constructs that help capture how people seek information, and to consolidate these various aspects, an adapted version of the Comprehensive Model of Information Seeking (CMIS) is used (Figure 1).^8^, ^9^

Factors that motivate (antecedents), one's background characteristics (demographics), how applicable or relevant the information need is (salience) and one's prior feelings regarding the need to seek information (beliefs) influence one to seek information.^8^ In the case of COVID‐19, trust in the information and healthcare system in relation to the intention to vaccinate^10^, ^11^, ^12^ is essential. Trust in vaccines information is key because the general public is often unable to fully assess the correctness of public health recommendations.^13^

Moreover, potentially more than ever before, during a global pandemic, people wish to be well‐informed and ask for more health‐related information.^14^ Important factors are, for example, users' perceptions of the information (characteristics). Previous studies show that people use different information sources, both offline and online, to access health‐related information.^15^, ^16^, ^17^, ^18^ Healthcare providers are currently still the most trusted source of information, though, seeking online information via the internet has gained popularity and has become an important information source to the public.^19^, ^20^ Seeking online information allows for direct answers, readily available and (mostly) accessible information in large quantities. Though this information may not be easily comprehensible to the wide public, the trustworthiness and objectivity of the information should also be examined carefully.^21^, ^22^ Due to the newness of the virus and uncertainties about the effectiveness of the vaccines, it is interesting to see where people who have had COVID‐19 seek or receive their COVID‐19 vaccine information and their perceptions of the quality of this information.

Lastly, information‐seeking actions such as whether one decides to vaccinate based on the sought or received information is important. Provision of information that meets the expectations of the public is key for those to decide to vaccinate, specifically trust that COVID‐19 vaccines are safe and effective.^2^, ^3^, ^23^ However, the most common reasons for hesitation or refusal to be vaccinated with the COVID‐19 vaccines were fear of side effects, safety and effectiveness.^24^, ^25^, ^26^, ^27^ It is therefore important to understand whether the provision of the current information on the vaccines is tailored in such a way that people who have had COVID‐19 decide to vaccinate.

Taking the different factors from the CMIS model into account, we investigated the characteristics of people who have had COVID‐19, that sought or received COVID‐19 vaccines information, where they sought the information, what they thought of the information, and which health information‐seeking behaviours and actions they took. The aim of this study was to understand the provision and need, quality of and trust in COVID‐19 vaccines information from the perspectives of people who have had COVID‐19 infection. This is a unique target group as their views on the vaccines may be different than those who have not had the virus.

## 2 METHODS

### 2.1 Study design

Nivel (Netherlands Institute for Health Services Research) has set up a cohort of people who have had COVID‐19 to provide insight into the course, severity and short‐ and long‐term consequences of COVID‐19. This panel design provides for a wide range and large sample of people reflecting the heterogenous patient population in daily general practitioner (GP) care practice. In this study, we conducted a secondary analysis of the cohort data.

### 2.2 Setting

This study took place in the period when the Netherlands experienced the third wave, and the alpha, followed by the delta variant, were the most prevalent.^28^ The participants in this study had COVID‐19 in the first half of 2021. At that point in time, about two million Dutch inhabitants had been diagnosed and registered with COVID‐19. Also, 32,438 people had died in the Netherlands with COVID‐19, either registered or probable cause of death.^28^ Further, during this time, the start of the country‐wide vaccination rollout took place.

### 2.3 Nivel Corona Cohort recruitment process

In Table 1, the different recruitment phases of GPs and patients for the Nivel Corona Cohort are described.

**Table 1:** Nivel Corona Cohort recruitment process

Recruitment phase	Activity
1. Eligible general practitioners to participate in study	In May 2020, GPs participating in Nivel Primary Care Database (Nivel‐PCD) were enquired to assess feasibility of the study. In total, 90 practices had shown interest in the study after a call from the research team. In February 2021, a selection of 25 GPs were invited via e‐mail to participate in this study. GPs were selected based on completeness of morbidity data in 2019, having delivered weekly data in 2020, using R83.03 to code COVID‐19 cases and having sufficient COVID‐19 cases.
2. Recruiting general practitioners to participate in study	Of the25 GPs invited to participate in the Nivel Corona Cohort, 18 practices participated. The selected GPs showed interest in participation, had sufficient quality and completeness of routinely registered electronic health record (EHR) data and had a sufficient number of COVID‐19 registrations.
3. Selecting patients from general practitioners to participate in study	People in the Nivel Corona Cohort were recruited from GPs that participate in the Nivel‐PCD and could only participate after invitation. Nivel‐PCD collects EHR data from around 500 GPs spread throughout the Netherlands. Nivel‐PCD receives data on a weekly basis from approximately 350 practices, with more than one million listed people, allowing to identify prevalent and incident COVID‐19 cases. Data in Nivel‐PCD is pseudonymized at the GP, Nivel does not receive directly identifying data such as name or address.^29^
4. Diagnosis of COVID‐19 for an individual patient	In the Netherlands, GPs use the International Classification of Primary Care to code the complaints and diseases their patients present to them. People with COVID‐19 were detected based on ICPC‐1 code R83.03 (COVID‐19). This ICPC‐code was introduced by the Dutch College of General Practitioners to register COVID‐19 as of November 2020. The diagnosis of COVID‐19 for an individual patient could be in their EHR when the patient consulted their GP directly, or when the patient contacted the Municipal Health services (GGD), who provided the national testing facilities. The GGD sent information on positive tests to GPs via automated coupling, using the R83.03 code, under the requirement that patients gave consent.
5. Generation of study pseudonym for each patient	A study pseudonym was generated for each patient, that allows for data linkage between Nivel‐PCD and the patient's filled in questionnaires. In Nivel‐PCD it is possible, but only via a trusted third party, to link the pseudonyms with a patient identification number that is known only in the practices' domain.^29^ This allowed us to initially flag eligible people for the Nivel Corona Cohort and to let GPs subsequently conduct a check on whether they were indeed eligible for participation.
6. Inclusion/exclusion of patients	People were excluded from this study if the GP indicated that the patient was not eligible to participate (i.e., due to the burden of filling in the questionnaire for this person or cognitive or personal problems hindering participation, or due to not being proficient in the Dutch language).
7. Invitation of patient through trusted third party	The GP provided names and addresses of suitable people to the trusted third party who invited people on behalf of their practice. The invitation contained the patient's study pseudonym.

Abbreviation: COVID‐19, coronavirus disease 2019.

### 2.4 Participants

If a patient decided to participate, they were asked to register online with the study pseudonym. During the registration process, the patient was asked for informed consent for study participation and data linkage. Once registered, the patient received invitations per e‐mail to questionnaires at enrolment and after three months.

Person inclusion for the Nivel Corona Cohort started in April 2021, and people were recruited both retrospectively and prospectively. At the start of the study, the GP invited all people with a known COVID‐19 infection in the past six weeks, and then every two weeks after the start of the study, newly diagnosed people were invited to participate.

### 2.5 Data collection

For this particular study, we used data from the two first online questionnaires sent to people in the Nivel Corona Cohort (Q1, the start of participation and Q2, at three months). Since we used data from both questionnaires (Q1 and Q2), we only included the people in this study who filled out both the first and second questionnaires. A reminder was sent after one week. People who did not respond or refused to participate in the first questionnaire were marked in the database and were not approached for further participation. We used data from people who filled in their second questionnaire (Q2) before the 28th of January 2022. All data were stored on Nivel's protected server. The questionnaire data was pseudonymized.

See Supporting Information: Appendix 1, for an overview of the questionnaire topics, outcome measures and types of responses possible, based on the CMIS. See Table 2 for the constructs of the adapted CMIS to fit the context of COVID‐19 vaccine information‐seeking behaviour.

**Table 2:** Study constructs in the context of the CMIS

CMIS construct	Description of measures
Antecedents	Background characteristics (Q1 questionnaire), first moment a participant filled in the questionnaire, were used.
	(1) gender (2) age (3) level of education (low, middle, high)
	*The categorization of the education level is in accordance with Statistics Netherlands (Low: primary education, prevocational secondary education (VMBO), Middle: senior general secondary education (HAVO), pre‐university education (VWO), senior secondary vocational education (MBO); High: higher vocational education (HBO) and university education (WO)*.^30^ In Q2, second filled in questionnaire, questions on health literacy (based on the Chew's Set of Brief Screening questions [SBSQ])^31^, ^32^, ^33^ were posed.
	Personal relevance factors (salience and beliefs) posed:
	(1) the degree in which people have trust in the sought/received information as well as trust in healthcare system. (2) how applicable or relevant the vaccines information was for them.
Information carrier factors	In the Q2 questionnaire, we collected specific information on the COVID‐19 vaccines information. All questions pertaining to COVID‐19 vaccines information included the information people actively sought themselves as well as the information they received from healthcare providers or other source. These two forms of receiving information (actively searching and passively receiving) are not split up in the questions asked.
	These questions included:
	(1) perceived quality of information, (2) whether the information was misleading or inaccurate, (3) type of information sources used to search for information on the COVID‐19 vaccines.
	See Supporting Information: Appendix 1, for the types of responses.
Information seeking action	Based on whether the people who have had COVID‐19 sought or received information (and which information), we asked whether the information met their expectations to make a choice to vaccinate (yes/no), and if no, an open question was posed about what they would have desired regarding the information provision.
Overall process*	The original CMIS shows the information‐seeking process as a linear process. The arrows, from left‐to‐right, in this model suggesting information seeking as a process that follows the factors from left‐to‐right. However, one can start and stop searching at different points based on beliefs and perceptions that may change over time. Hence, in the adapted model in this study (Figure 1), there are no arrows, but instead the factors presented with each important CMIS theme (antecedents, information carriers and actions).

Abbreviations: CMIS, Comprehensive Model of Information Seeking; COVID‐19, coronavirus disease 2019.

### 2.6 Data analysis

We used descriptive analyses (frequencies [*N*, %], means [SD]) to describe the population and outcomes. For the personal factors, we looked at differences in age, gender, level of education, health literacy, vaccination status, (in)sufficient trust in the COVID‐19 vaccination information, and trust in the healthcare system in relation to the outcomes. The outcomes included were: whether (and where) the person sought/received information, perceptions of information quality, whether the information was misleading or inaccurate, and whether the information was sufficient/met the person's expectations to make a well‐informed decision to vaccinate.

The differences were analysed using analysis of variance tests (F‐tests) and *χ* ^2^ tests. We chose group comparisons as this is an explorative study looking at different facets of information‐seeking behaviour and how this differs amongst different types of COVID‐19 people (e.g., younger vs. older people, higher educated vs. lower educated). Tukey post hoc tests revealed the difference in means in the groups of respondents with different background characteristics. A significance level of *p* < .05 was used. We used the statistical software STATA version 16 for the analysis of the questionnaires.

## 3 RESULTS

We invited 1851 people for the Nivel Corona Cohort. In total, 442 people filled in the Q1 questionnaire, at the start of their participation in this study. Of these respondents, 314 also filled in the Q2 questionnaire (net response rate 70%).

### 3.1 COVID‐19 exposure and vaccination

All people have had COVID‐19, of which many (58%) had the virus one–three months before filling in the Q1 questionnaire. The majority (89%) of the respondents received the COVID‐19 vaccines after they had COVID‐19. Over the period of three months (Q1 and Q2), there was an increase in the number of respondents vaccinated with at least one vaccine (68%–96%). An overview of sample characteristics is reported in Table 3.

**Table 3:** Background characteristics of sample population

Characteristics	*N* (%)
Age (*N* = 311) (years)	
<40	33 (11)
40–64	234 (75)
≥65	44 (14)
Gender (*N* = 314)	
Male	118 (38)
Female	196 (62)
Country of birth (*N* = 307)	
The Netherlands	292 (96)
Other	15 (4)
Education (*N* = 290)	
Low	35 (12)
Middle	130 (45)
High	125 (43)
Health literacy score (*N* = 314)	
Adequate health literacy	309 (98)
Inadequate health literacy	5 (2)
Vaccination status (*N* = 311)	
Vaccinated with at least one vaccine	298 (96)
Not (yet) vaccinated	13 (4)
Trust in COVID‐19 vaccines information (*N* = 274)	
Sufficient	245 (89)
Insufficient	29 (11)
Trust in healthcare system (*N* = 297)	
Sufficient	278 (94)
Insufficient	19 (6)

Abbreviation: COVID‐19, coronavirus disease 2019.

### 3.2 Adapted CMIS for COVID‐vaccines related information

**Figure 2:**
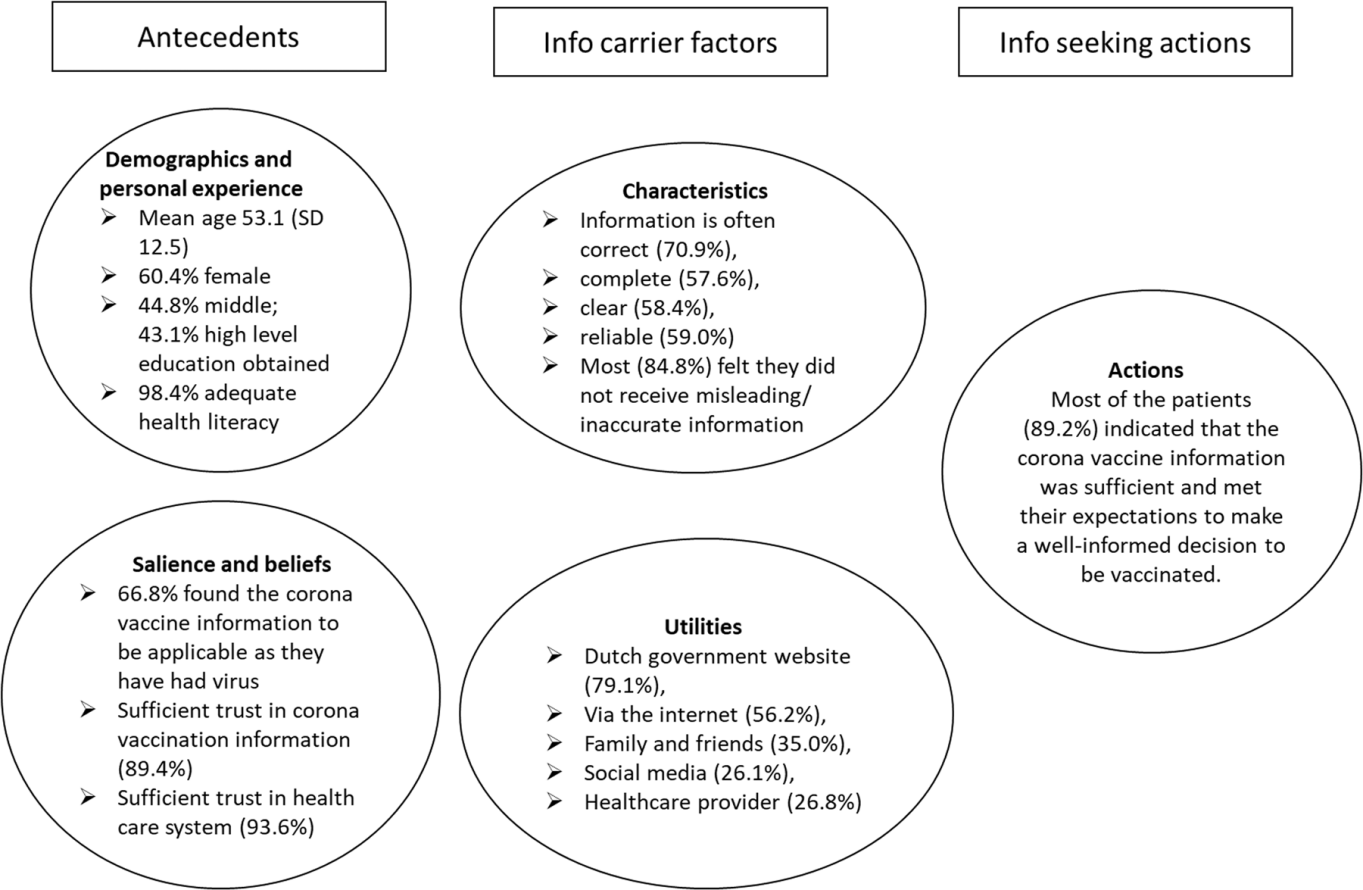
Adapted Comprehensive Model of Information Seeking to information seeking behaviour for COVID vaccines related information based on the experiences of people who have had COVID‐19. COVID‐19, coronavirus disease 2019.

The factors that influence information‐seeking behaviour of people with COVID‐19 selected in this study are presented according to the CMIS, as shown in Figure 2.

#### 3.2.1 Antecedents

Most people were female (62%), obtained a middle (45%) or high level (43%) of education, and were middle‐aged (mean: 53.1, SD: 12.5). The majority had a self‐perceived adequate health literacy (98%), implying that the role of health literacy cannot further be analysed as the number of respondents with an inadequate health literacy score was too small (Figure 2, *antecedents*).

#### 3.2.2 Personal relevance factors (salience and beliefs)

About two‐thirds of the people (67%) found the information provided or sought on the COVID‐19 vaccines to be applicable to them as they have had the COVID‐19 virus, specifically people who have more trust in the information (*p* < .0001) and those with more trust in the healthcare system (*p* < .0001).

Moreover, the respondents who indicated that the information was applicable to them mainly indicated in an open question that the information they sought was complete, clear and accurate. They also indicated that the information was applicable to their situation as they had only needed one vaccination as they are people who have had COVID‐19. Those who did not think the information was applicable to them indicated in an open question that the information was too general and could be applicable to all people (not specific to those who have had the virus, that is, when to vaccinate after the recovery period), that the side effects, long‐term effects and effect of natural immunity against the virus were not clearly indicated as much information had not been based on scientific evidence, and/or that some information is contradictory (i.e., one or two vaccines necessary for people who have had COVID‐19). Lastly, the majority of the respondents (89%) had a sufficient amount of trust in the information on the COVID‐19 vaccines and trust in the healthcare system (94% of respondents).

### 3.3 Information carrier factors

#### 3.3.1 Perceptions on information quality

Respondents were asked to assess the quality of the COVID‐19 vaccines information (Table 4). The majority, 71%, of the respondents often found the information to be correct. Fewer respondents often found the information reliable (59%) or clear (58%). More than half (58%) indicated that the information was often complete, and two‐thirds of the respondents, 67%, sometimes found the information to be contradictory. Also, respondents were inquired how applicable they perceived the information to be for their situation. Less than half, 41%, indicated this to be the case.

**Table 4:** People who had COVID‐19 perceptions on the information they received/sought about the COVID‐19 vaccines

	Always	Often	Sometimes	Never
The information is correct (*N* = 295), *N* (%)	23 (8)	209 (71)	63 (21)	‐
The information is complete (*N* = 297), *N* (%)	18 (6)	171 (58)	101 (34)	7 (2)
The information is clear (*N* = 298), *N* (%)	27 (9)	174 (58)	93 (31)	4 (1)
The information is reliable (*N* = 300), *N* (%)	37 (9)	177 (59)	94 (31)	2 (1)
The information is up to date (*N* = 299), *N* (%)	26 (9)	177 (59)	91 (30)	5 (2)
The information applies to me (*N* = 298), *N* (%)	13 (4)	122 (41)	155 (52)	8 (3)
The amount of information is just right (*N* = 298), *N* (%)	11 (4)	152 (51)	125 (42)	10 (3)
Information from different sources contradicts each (*N* = 293), *N* (%)	13 (4)	62 (21)	196 (67)	22 (8)

Abbreviation: COVID‐19, coronavirus disease 2019.

#### 3.3.2 Perceptions on information quality in relation to background

People with different background characteristics had varying perceptions of information quality. Primarily, higher educated people, those who had sufficient trust in the COVID‐19 information and in the healthcare system, and who were younger (primarily <40 years old) were more positive about the information quality, see Supporting Information: Appendix 2.

#### 3.3.3 Misinformation regarding the COVID‐19 vaccines

Most (85%) of the respondents (*N* = 277) felt that they did not receive misleading or inaccurate information regarding the COVID‐19 vaccines. The respondents (*N* = 42) who did find the information on the COVID‐19 vaccines to be misleading or inaccurate, mentioned various reasons in an open question. People who found the information misleading or inaccurate, specifically indicated the information about the side effects (*N* = 3), (long‐term) consequences, safety in use and effectiveness of the vaccines (*N* = 9), but also regarding the related deaths (*N* = 2). Also, people found the quality of the information poor (i.e., not reliable, misleading, inconsistent, contradictory, incomplete and/or not based on enough scientific evidence) (*N* = 10). Some found the information based too much on political views (*N* = 3), not suited for those who have already had the virus and the effects of natural immunity (*N* = 3) and based on conspiracies and those who are against vaccinating (*N* = 3).

#### 3.3.4 Information source about the COVID‐19 vaccine

The most used information source was the central Dutch site on COVID‐19 vaccines information, the National Institute for Public Health and the Environment (RIVM) website (Table 5). The majority of the respondents (79%) sought or received information about the COVID‐19 vaccines on this website. Additionally, respondents sought or received information on the internet via different search engines (56%), via family and friends (35%), social media (26%) and healthcare providers (27%). About one‐quarter of the respondents (22%) used the Dutch website with primary care information from the GP (thuisarts.nl). Lastly, a minority of the respondents searched for information on different websites (13%).

**Table 5:** Information sources where respondents found information on the COVID‐19 vaccine

Information source	*N* (%)
On the central Dutch site on COVID‐19 vaccination information	242 (79)
On the internet via a search engine, such as Google, Bing or Yahoo	172 (56)
With family/friends/acquaintances	107 (35)
On social media	80 (26)
With a doctor or other healthcare provider	82 (27)
Other	29 (10)
On the website of Thuisarts.nl	66 (22)
On another website	39 (13)
At a hospital	27 (19)
In a patient organization	10 (3)
With a health insurer	2 (1)
I did not seek nor receive information	20 (7)

Abbreviation: COVID‐19, coronavirus disease 2019.

Primarily younger, higher educated, females were more likely to seek information on the RIVM website. Generally, older people and lower educated sought information on social media. For other comparisons of where people sought or received information and their background characteristics, see Supporting Information: Appendix 3.

Those who did not seek nor receive information (7%) gave various reasons. Some indicated that they did not see the added value (*N* = 2) nor the need (*N* = 5). Others indicated that there was already enough presented in the media (*N* = 1), or specifically in the newspaper (*N* = 1). Some respondents already had enough trust in the information or in others/faith in science or had enough information (*N* = 2). Some expressed being tired of the situation (*N* = 3). Others mentioned they were already informed or their situation was clear (*N* = 5).

### 3.4 Information seeking action

#### 3.4.1 Information provision and decision to vaccinate

Most of the people (89%), on the whole, agreed with the question about how sufficient the information was and whether this met their expectations, and whether to make a well‐informed decision to vaccinate. People with sufficient trust (*p* < .0001) in the information on the vaccines and in the healthcare system (*p* < .0001) were more likely to believe that the information provided on the COVID‐19 vaccines was sufficient to make a well‐informed decision to vaccinate. At the same time, about one‐third (32%) indicated that the information was sometimes‐to‐never reliable (41%) nor clear (32%).

In case the information was not sufficient (*N* = 30), respondents gave the following points that could have been better with regard to the provision of information: more honesty/transparency, such as information based on facts and research, and the need for the potential consequences and disadvantages of the vaccines to be more openly discussed. Also, there is a desire for the pros and cons to be made more explicit. Moreover, respondents indicated that quite a lot is still unknown, and some respondents would want less emphasis on only the positive elements, and instead more transparency. Lastly, some expressed the need for more clarity and clear communication, for example, why the AstraZeneca vaccine was mainly limited to the 60–64‐year‐old group and what the added value is in comparison to natural immunity.

## 4 DISCUSSION

The sample population who sought or received information about the COVID‐19 vaccines on a major governmental‐related website for this, via the internet, or via family and friends. Most people found the information correct and did not perceive the information to be misleading, while fewer people found the information reliable and clear.

Revisiting the CMIS model, the antecedents are important factors to start the information‐seeking process. These are often motivators to start the search process, which can reduce uncertainty, confirm/disprove certain beliefs or ideas, or satisfy curiosity.^34^ While, most people were vaccinated with at least one vaccine at the moment of filling the second (Q2) questionnaire, we cannot conclude on the motivations to vaccinate. It is also important to note here that it is not necessarily everyone's decision to only take a vaccine after infection, but that this also has to do with the fact that vaccinations only started then. Nevertheless, we can comment on characteristic types of people who used which information sources and what their perceptions were on the (quality of the) information during their information‐seeking process.

Certain types of people were more inclined to seek health information, such as women, those higher educated and younger people.^35^, ^36^, ^37^ This also is in line with our sample population, primarily younger, higher educated, females and those who had sufficient trust in the healthcare system were more likely to seek information on the RIVM website, who sought information on the Dutch government website, a trustworthy and reliable website. The representativeness of the cohort was checked (unpublished manuscript, can be requested by the corresponding author). Comparisons were made between the Nivel Corona Cohort and the groups of selected and invited patients that did not participate. The Nivel Corona Cohort particularly included specific types of people, generally older and more often female.

Trust, an important antecedent, is also a motivator to seek information. A large group of the respondents had trust in the COVID‐19 vaccines information and healthcare system, while about 1 in 10 found the information misleading and inaccurate. Based on the Integrative Model of Organizational, trustworthy information can be defined with traits such as technical knowledge, widely open and accessible, factual, reliable and consistent.^38^ Lack of trust is also often associated with perceptions that the information is misleading or inaccurate. Also, this was shown in this study, in the partly tautological relationships (e.g., people who had less trust in the information were more likely to agree that information was misleading or inaccurate) as the three central dimensions of trust are benevolence, integrity and ability, which are related to accuracy.^38^

Moreover, during the information search process, the antecedent factors can still cause an individual to stop their search. Reasons for this may be that they become overwhelmed by the information. This is why it is important to enquire about the perceptions of information quality. Overall, the respondents found the information to be applicable and complete, whilst, there were also respondents (more than half) who found the information to be contradictory on an occasional basis, and less reliable or clear. Evaluating one's perceptions of the quality of the information is a challenge, as these are based on subjective perspectives. However, these factors do act as a starting point to further enquire about what changes the information‐seekers' (or lack thereof) needs and desires in terms of patient‐tailored information. For example, despite the small proportion of the respondents who found the information misleading, or not applicable to them, we can follow up on their suggestions that the information could have been more transparent and more clear. Specifically, information can be more clear on whether people who have had the virus need one or two vaccine(s), what the long‐term consequences are of the vaccine and the effects (i.e., herd immunity and build‐up of antibodies) of the vaccines. Evidence shows that transparent communication may harm vaccine acceptance here and there, however, transparency increases trust in health authorities.^29^ On the contrary, vague and sometimes reassuring communication does not increase vaccine acceptance either. Ambiguity in communicating information may lead to lower trust and higher endorsement of the spread of misinformation.^27^ In return, to vaccinate large amounts of people, it is crucial that people trust in the fact that COVID‐19 vaccines are safe and effective,^2^, ^3^, ^23^ as well as the effectiveness of boosters in the future.^5^

Lastly, information‐seeking action, the majority of the study sample population indicated that the information about the COVID‐19 vaccines was sufficient. Also, the information met their expectations to make a well‐informed decision about whether or not to be vaccinated. For those who indicated no, we also enquired the reasons why, which in return helps to tailor the needs of people who both actively search for information and the group who prefers not to. Reasons why people do not seek information or avoid information are because information can conflict with their prior knowledge, beliefs and attitudes, or potentially causes heightened emotion such as anxiety or stress about the information.^39^

Situations where uncertainty is present can cause increased anxiety and risk perception^40^, ^41^ amongst individuals seeking healthcare/treatment. As a result, this can decrease well‐informed and optimal healthcare decisions as well as avoidance behaviours.^40^, ^41^ In the case of COVID‐19, this is a unique situation, because there was more uncertainty regarding how fast the vaccines were developed, and there was an increased level of infection‐related uncertainty, in the context of a global pandemic.

Due to rapidly emerging vaccines and without, at the time of development, sufficient evidence as to their effectiveness and health impact,^40^ this can cause uncertainty for people to vaccinate. Our study adds the perspective of people who have already had COVID‐19 and their perceptions of the COVID‐19 vaccines information, and whether this influences their decision to vaccinate. Their perceptions may be different to those who did not have an infection yet and choose to vaccinate as they had already had COVID‐19, including views on risk‐perception of the virus/need to vaccinate.

An important way to decrease the spread of the virus is by mass vaccination uptake.^2^ Though, to realize this, people should be well‐informed and feel confident to make a choice to vaccinate. Solely providing information about COVID‐19 vaccines is not sufficient. More importantly, the information should be tailored to the needs of the people seeking vaccine information. One of those elements was whether people who already had had a COVID infection and therefore might have other questions or information needs regarding vaccination than those who did not experience an infection. To be able to tailor information, it is important to be aware of the perceptions of and the trust in the information of different groups, and our study contributes to insights into the perceptions of those who already experienced an infection. The people with COVID‐19 that found the information misleading/or inaccurate indicated that this was generally due to safety and efficacy reasons related to the vaccines. This is in line with research conducted in the United Kingdom (Freeman et al.^42^) whereby people who were strongly hesitant towards the vaccine were less likely to see the collective benefit^42^, ^43^ of vaccination, and instead had more concerns about the safety and fast development of the vaccines. A way to manage uncertainty in healthcare is by communicative practices, whereby the information moderates the effect of the uncertainty.^44^ Reliable and accurate information, as well as information about the safety and effectiveness of the vaccines, can decrease the uncertainty about whether to vaccinate, also in those who already had an infection.

Over time, the need for effective communication strategies for uncertain healthcare‐related situations has increased, as well as the nature of uncertain situations has become more complex. Therefore, there is a need to know how to tailor information to accommodate this uncertainty.^45^ The lessons learned from this study give room to further tailor information about vaccines in future pandemics or vaccination campaigns. The communication strategies (i.e., using the perceptions about the quality and how/where people seek information about vaccines sought/received in those who might already feel immunized because they experienced an infection) are important to more accurately target patients about vaccines in future vaccination campaigns, booster programs or pandemics. Based on our results, to make sure to tailor the information to their needs, respondents indicated that more honesty and transparency in the information is needed, that information is backed up by facts and research, and there is more overall clarity in the communication and information (e.g., whether people need one or two vaccine(s) if they have had the virus).

### 4.1 Strengths and limitations

One of the strengths of this study included the varied sample of people, reflecting the general population. We recruited people from 18 practices, and people varied in the severity of COVID‐19. We also included those with mild complaints. Another strength was using participants drawn from practices, providing a well‐defined population, in which COVID‐19 has really been diagnosed. If not drawn from a practice, then the sample population would have consisted of self‐reported diagnoses.

An important limitation is the small number of people who had not had a vaccine in the sample. In our study, we have a relatively large number of people who were vaccinated, hence we cannot draw conclusions about unvaccinated people. The moment of the questionnaire could have played a role. In hindsight, including the information on COVID‐19 vaccines questions in Q1 and Q2, both before and after being vaccinated, would have been more appropriate. Now we could only report on the group of people who have primarily already been vaccinated. According to the Dutch governmental website on COVID‐19 vaccination, 89% of people aged 18 and older have now had at least one COVID‐19 vaccine.^46^ In our sample, the mean age was 53 years old, and people over 55 or 65 remain the most receptive group to vaccination, which may explain the high vaccination rate.^26,47^

Another limitation is the small group of people with inadequate health literacy (*N* = 5), suggesting we could not study this sample. It is important to note that not all low‐educated people have inadequate health literacy, as shown in our sample, only one person indicated inadequate self‐reported health literacy and a low obtained education level. The small group of people with inadequate health literacy may be related to the ease or difficulty that people with a lower health literacy may experience when completing questionnaires. In our study, we also had a large group of people with, on average, a high education level. Amongst various potential reasons why, one particular reason why this may not have such a large role is that there is not a big difference in vaccination coverage between low, middle and highly educated people in the Netherlands. According to Statistics Netherlands, about three‐fourths (77%) of the highly educated people 25 years and older indicated that they intend to vaccinate, while slightly fewer low educated (68%) and people who obtained a middle‐level education (69%).^48^

Additionally, a large part of the questions is based on single‐item and self‐reported measures. We asked people who have had COVID‐19 what they think of the COVID‐19 vaccines information. The information we collected (whether something is reliable, clear or trustworthy) is someone's opinion, however, it could be that these people read misleading information and did not realize it. Our approach was to see whether people have the feeling that the information is adequate and correct, and not whether this was actually the case. For example, we did not visit the information sources to test the accuracy or the trustworthiness of the information.

Lastly, participants were not explicitly asked about the information content they sought/received. They were only asked which information source they used to seek/receive COVID‐19 vaccines information and their perceptions of the information.

#### 4.1.1 Clinical implications

Tailoring COVID‐19 vaccines information to specific people's characteristics, and increasing clarity and transparency are important for accommodating the information needs of different types of people. Primarily younger, higher educated, females and those who had sufficient trust in the healthcare system were more likely to seek information on the Dutch government website, a reliable and trustworthy source. More attention should go out to set up ways to make the COVID‐19 vaccines information provision more inclusive, for example, males, lower‐educated people, and those that have less trust in the government/healthcare system. The reasons why people had (or lacked) trust in the information about vaccines and the healthcare system, as well as motives to vaccinate, could also be further investigated. Also, while we collected data on migration background, it proved that the vast majority of participants were of Dutch descent. Migration background might be an interesting aspect in relation to vaccine uptake decisions and trust in the vaccine for a future study. Potentially selecting GPs in communities with a higher population of migrants may result in a more heterogenous and diversified study population. These lessons learned can increase effective communicative strategies in future pandemics, vaccination campaigns or booster programs.

## 5 CONCLUSION

Different characteristics of people who had COVID‐19 and sought information were identified, which is important to offer tailored information. Among this vaccinated, and generally higher educated, middle‐aged, female population, people who had COVID‐19 were generally positive about the vaccines information, but overall the reliability and clarity could be improved. Reliable and clear information is important for a high vaccination uptake; for other vaccine programs, including booster programs and coming pandemics. More research is necessary to draw conclusions on the perceptions of the COVID‐19 vaccine information in the group of unvaccinated people.

## AUTHOR CONTRIBUTIONS

Laura Schackmann, Karin Hek and Liset van Dijk contributed to the conceptualization and design of this project. Data analysis was led by Laura Schackmann under the supervision of Karin Hek. Data interpretation and a critical review of the results were discussed with all the authors. Laura Schackmann wrote the first draft of the paper under the supervision of Liset van Dijk. All authors contributed to reviewing and editing subsequent drafts and reviewed the final manuscript.

## CONFLICTS OF INTEREST

L. v. D., M. V. and L. S. received funding from TEVA Pharmaceuticals for a study not related to this study. L. v. D. and M. V. also received funding from AstraZeneca for a study not related to this study. The remaining authors declare no conflict of interest.

## ETHICS STATEMENT

The Medical Ethics Committee (METc) of the Vrije University Academic Medical Centre (VUMC) approved the protocol (METC protocol number 2020.0709). The METc VUMC concluded that this study is not a clinical research with human subjects as meant in the Medical Research Involving Human Subjects Act (WMO). The study was also approved according to the governance code of Nivel‐PCD under number: NZR‐003120.081. All participants gave informed consent before starting the questionnaire. Participation in the study included giving permission to link data from the questionnaire to the Nivel‐PCD data of this person. The anonymity of all respondents is guaranteed.

## Supporting information

Supporting information.

Supporting information.

Supporting information.

## Data Availability

The data that support the findings of this study are available from the corresponding author upon reasonable request.
