# Supplementary material for: Provision of and trust in COVID‐19 vaccines information: Perspectives of people who have had COVID‐19
Source: Health Expect. 2023 Feb 3;26(2):806–17. doi: 10.1111/hex.13706 (PMC10010094; doi:10.1111/hex.13706)
Supplement: Supplementary file 1 — Supporting information. [file HEX-26--s002.docx]

Appendix 1.

Table A. Overview of the questionnaire topics, outcome measures, and types of responses possible, based on the Comprehensive Model of Information Seeking (CMIS).

| **Outcome measures/ questions** | **Type of responses** | **Originates from Q1 or Q2** |
| --- | --- | --- |
| **COVID-19 exposure and vaccination** | | |
| How many months prior to filling out the questionnaire the person had the virus | shorter than one month ago, one-three months ago, four-six months ago, longer than six months ago, I do not/do not want to answer | Q1 |
| Vaccination status | whether the person is vaccinated with at least one vaccine (yes/no) | Q1 |
| **CMIS topics** | | |
| *Antecedents** | | |
| *Background* | | |
| Gender | Male/female | Q1 |
| Age | <40 years old, 40-64, 65 and older | Q1 |
| Level of education | low, middle, and high level | Q1 |
| Health literacy | Chew’s Set of Brief Screening questions (SBSQ) use used: the respondent’s health literacy score was calculated by taking the sum of the three five-point Likert scale questions, a scale from zero to four; the average score of the three questions was taken^30^. An average score of two or less indicates inadequate health literacy, and a score above two indicates adequate health literacy^31-32^ | Q2 |
| *Salience and beliefs* | | |
| Degree in which people have trust in the read/received information as well as trust in healthcare system | sufficient trust: greater than or equal to six, report score one-to-ten; insufficient trust: less than six, report score one-to-ten | Q2 |
| How applicable or relevant the vaccines information was (yes, because and no, because) | yes, because…  no, because…  *The why answers were derived from open questions.* | Q2 |
| *Information carrier factors* | | |
| Perceived quality of information (i.e. how often the person found the information to correct, complete, clear, reliable, up to date, applicable, just right, and whether the information from different sources contradicted each other). | four-point scale (never, sometimes, often, always) | Q2 |
| Whether the information was misleading or inaccurate | (yes/no) | Q2 |
| What the respondent found misleading about the information. | Open answer | Q2 |
| Type of information sources used to search for information on the COVID-19 vaccines (i.e. via family/friends, social media, healthcare providers, via the internet, at a hospital, or from other sources such as a patient organization or health insurer.). | yes/no | Q2 |
| *Information seeking action* | | |
| Whether the information met the expectations of these people to make a choice to vaccinate | Yes/ no | Q2 |
| If answered no on the above-mentioned question, respondents were asked what they would have desired regarding the information provision | Open question | Q2 |

* In the Q1 questionnaire several topics were asked, many not used in this study, such as: complaints experienced by COVID-19 over time and their severity, (self) care, lifestyle, quality of life, impact of COVID-19, social support, information provision on the COVID-19 virus, and understanding of health information and health literacy.
