# Supplementary material for: Provision of and trust in COVID‐19 vaccines information: Perspectives of people who have had COVID‐19
Source: Health Expect. 2023 Feb 3;26(2):806–17. doi: 10.1111/hex.13706 (PMC10010094; doi:10.1111/hex.13706)
Supplement: Supplementary file 2 — Supporting information. [file HEX-26--s003.docx]

Appendix 2.

Table B. Comparisons between background characteristics and perceived quality of information

|  | Contrast, mean (SD) | p-value |
| --- | --- | --- |
| Up-to-date information |  |  |
| Education level |  | F (2, 281) = 3.0, p=0.05 |
| *Low* | 2.6 (.7) |  |
| *Middle* | 2.7 (.6) |  |
| *High* | 2.9 (.6) |  |
| Trust in the vaccines information |  | F (1, 259) = 15.4, p=0.0001 |
| *Sufficient trust* | 2.8 (.6) |  |
| *Insufficient trust* | 2.3 (.7) |  |
| **Correct information** |  |  |
| Education level |  | F (2, 277) = 5.1, p<.01 |
| *Low* | 2.7 (.6) |  |
| *Middle* | 2.8 (.4) |  |
| *High* | 3.0 (.5) |  |
| Trust in the vaccines information |  | F (1, 255) = 14.4, p<0.001 |
| *Sufficient trust* | 2.9 (.5) |  |
| *Insufficient trust* | 2.5 (.5) |  |
| Trust in healthcare system |  | F (1, 278) = 3.9, p=0.05 |
| *Sufficient trust* | 2.9 (.5) |  |
| *Insufficient trust* | 2.6 (.5) |  |
| **Reliable information** |  |  |
| Education level |  | F (2, 282) = 4.3, p<0.05 |
| *Low* | 2.6 (.7) |  |
| *Middle* | 2.7 (.5) |  |
| *High* | 2.9 (.7) |  |
| Trust in the vaccines information |  | F (1, 261) = 31.0, p<0.0001 |
| *Sufficient trust* | 2.8 (.6) |  |
| *Insufficient trust* | 2.2 (.6) |  |
| Trust in healthcare system |  | F (1, 284) = 9.5, p<0.01 |
| *Sufficient trust* | 2.7 (.6) |  |
| *Insufficient trust* | 2.3 (.6) |  |
| **Complete information** |  |  |
| Trust in the vaccines information |  | F (1, 257) = 13.4, p<0.001 |
| *Sufficient trust* | 2.7 (.6) |  |
| *Insufficient trust* | 2.3 (.7) |  |
| Trust in healthcare system |  | F (1, 280) = 4.5, p<0.05 |
| *Sufficient trust* | 2.7 (.6) |  |
| *Insufficient trust* | 2.3 (.6) |  |
| Age |  | F (2, 291) = 13.4, p<0.05 |
| *<40* | 2.8 (.8) |  |
| *40-64* | 2.7 (.6) |  |
| *≥65* | 2.5 (.7) |  |
| **Clear information** |  |  |
| Trust in the vaccines information |  | F (1, 259) = 16.0, p=0.0001 |
| *Sufficient trust* | 2.8 (.6) |  |
| *Insufficient trust* | 2.3 (.6) |  |
| **Found the information to be contradictory** |  |  |
| Age |  | F (2, 287) = 4.7, p<0.01 |
| *<40* | 2.2 (.7) |  |
| *40-64* | 2.2 (.6) |  |
| *≥65* | 2.5 (.8) |  |
| Trust in the vaccines information |  | F (1, 255) = 20.0, p<0.0001 |
| *Sufficient trust* | 2.2 (.6) |  |
| *Insufficient trust* | 2.7 (.8) |  |
| Trust in healthcare system |  | F (1, 276) = 4.4, p<0.05 |
| *Sufficient trust* | 2.2 (.6) |  |
| *Insufficient trust* | 2.5 (.9) |  |
| **Just right amount of information** |  |  |
| Age |  | F (2, 292) = 4.2, p<0.05 |
| *<40* | 2.7 (.7) |  |
| *40-64* | 2.6 (.6) |  |
| *≥65* | 2.3 (.8) |  |
| Trust in vaccines information |  | F (1, 258) = 9.4, p<0.01 |
| *Sufficient trust* | 2.6 (.6) |  |
| *Insufficient trust* | 2.2 (.6) |  |
| Trust in healthcare system |  | F (1, 281) = 5.6, p<0.05 |
| *Sufficient trust* | 2.6 (.6) |  |
| *Insufficient trust* | 2.2 (.6) |  |
| **Information to be more applicable to them** |  |  |
| Age |  | F (2, 293) = 3.7, p<0.05 |
| *<40* | 2.7 (.7) |  |
| *40-64* | 2.5 (.6) |  |
| *≥65* | 2.3 (.7) |  |
| Trust in vaccines information |  | F (1, 259) = 11.4, p<0.001 |
| *Sufficient trust* | 2.5 (.6) |  |
| *Insufficient trust* | 2.1 (.6) |  |
