# Supplementary material for: Provision of and trust in COVID‐19 vaccines information: Perspectives of people who have had COVID‐19
Source: Health Expect. 2023 Feb 3;26(2):806–17. doi: 10.1111/hex.13706 (PMC10010094; doi:10.1111/hex.13706)
Supplement: Supplementary file 3 — Supporting information. [file HEX-26--s001.docx]

Appendix 3.

Table C. Comparisons between background characteristics and where the people who had COVID-19 in this study sought or received COVID-19 vaccines information

|  | Contrast | p-value |
| --- | --- | --- |
| Sought information on the RIVM website |  |  |
| Gender, n(%) |  | χ² (1) = 5.0, p<0.05 |
| *Male* | 84 (72) |  |
| *Female* | 158 (83) |  |
| Education level, n(%) |  | χ² (1) = 15.5, p<0.0001 |
| *Low* | 20 (57) |  |
| *Middle* | 103 (79) |  |
| *High* | 109 (87) |  |
| Age, n(%) |  | χ² (2) = 27.4, p<0.0001 |
| *<40* | 27 (87) |  |
| *40-64* | 191 (83) |  |
| *≥65* | 21 (49) |  |
| **Sought information on social media** |  |  |
| Education level, n(%) |  | χ² (2) = 11.3, p<0.01 |
| *Low* | 14 (40) |  |
| *Middle* | 39 (30) |  |
| *High* | 20 (16) |  |
| Age, n(%) |  | χ² (1) = 6.8, p<0.01 |
| *<40 and 40-64* | 60 (23) |  |
| *≥65* | 18 (42) |  |
| **Sought information on the website of the Dutch website with primary care information from the general practitioner (thuisarts.nl)** |  |  |
| Education level, n(%) |  | χ² (1) = 3.9, p=0.05 |
| *Low and middle* | 29 (18) |  |
| *High* | 34 (27) |  |
| **Consult family and friends for information** |  |  |
| Trust in the vaccines information, n(%) |  | χ² (1) = 5.9, p<0.05 |
| *Sufficient trust* | 81 (34) |  |
| *Insufficient trust* | 16 (57) |  |
